# Perinatal Bisphenol A Exposure Induces Chronic Inflammation in Rabbit Offspring via Modulation of Gut Bacteria and Their Metabolites

**DOI:** 10.1128/mSystems.00093-17

**Published:** 2017-10-10

**Authors:** Lavanya Reddivari, D. N. Rao Veeramachaneni, William A. Walters, Catherine Lozupone, Jennifer Palmer, M. K. Kurundu Hewage, Rohil Bhatnagar, Amnon Amir, Mary J. Kennett, Rob Knight, Jairam K. P. Vanamala

**Affiliations:** a Department of Plant Science, The Pennsylvania State University, University Park, Pennsylvania, USA; b Animal Reproduction and Biotechnology Laboratory, Department of Biomedical Sciences, Colorado State University, Fort Collins, Colorado, USA; c Max Planck Institute for Developmental Biology, Tübingen, Germany; d Department of Medicine, University of Colorado Anschutz Medical Campus, Aurora, Colorado, USA; e Department of Food Science, The Pennsylvania State University, University Park, Pennsylvania, USA; f Departments of Pediatrics and Computer Science and Engineering, University of California San Diego, La Jolla, California, USA; g Department of Veterinary and Biomedical Sciences, The Pennsylvania State University, University Park, Pennsylvania, USA; h Center for Microbiome Innovation, University of California San Diego, La Jolla, California, USA; i The Penn State Hershey Cancer Institute, Hershey, Pennsylvania, USA; j Center for Molecular Immunology and Infectious Diseases, The Pennsylvania State University, University Park, Pennsylvania, USA; Oregon State University

**Keywords:** amino acid metabolism, endocrine disruptor, gut permeability, inflammation, lipopolysaccharide, microbiome, perinatal, rabbit, short-chain fatty acids

## Abstract

Bisphenol A (BPA) accumulates in the maturing gut and liver *in utero* and is known to alter gut bacterial profiles in offspring. Gut bacterial dysbiosis may contribute to chronic colonic and systemic inflammation. We hypothesized that perinatal BPA exposure-induced intestinal (and liver) inflammation in offspring is due to alterations in the microbiome and colonic metabolome. The 16S rRNA amplicon sequencing analysis revealed differences in beta diversity with a significant reduction in the relative abundances of short-chain fatty acid (SCFA) producers such as *Oscillospira* and *Ruminococcaceae* due to BPA exposure. Furthermore, BPA exposure reduced fecal SCFA levels and increased systemic lipopolysaccharide (LPS) levels. BPA exposure-increased intestinal permeability was ameliorated by the addition of SCFA *in vitro*. Metabolic fingerprints revealed alterations in global metabolism and amino acid metabolism. Thus, our findings indicate that perinatal BPA exposure may cause gut bacterial dysbiosis and altered metabolite profiles, particularly SCFA profiles, leading to chronic colon and liver inflammation.

**IMPORTANCE** Emerging evidence suggests that environmental toxicants may influence inflammation-promoted chronic disease susceptibility during early life. BPA, an environmental endocrine disruptor, can transfer across the placenta and accumulate in fetal gut and liver. However, underlying mechanisms for BPA-induced colonic and liver inflammation are not fully elucidated. In this report, we show how perinatal BPA exposure in rabbits alters gut microbiota and their metabolite profiles, which leads to colonic and liver inflammation as well as to increased gut permeability as measured by elevated serum lipopolysaccharide (LPS) levels in the offspring. Also, perinatal BPA exposure leads to reduced levels of gut bacterial diversity and bacterial metabolites (short-chain fatty acids [SCFA]) and elevated gut permeability—three common early biomarkers of inflammation-promoted chronic diseases. In addition, we showed that SCFA ameliorated BPA-induced intestinal permeability *in vitro*. Thus, our study results suggest that correcting environmental toxicant-induced bacterial dysbiosis early in life may reduce the risk of chronic diseases later in life.

## Abstract (precis)

Emerging evidence suggests that environmental toxicants may influence inflammation-promoted chronic disease susceptibility during early life. BPA, an environmental endocrine disruptor, can transfer across the placenta and accumulate in fetal gut and liver. However, underlying mechanisms for BPA-induced colonic and liver inflammation are not fully elucidated. In this report, we show how perinatal BPA exposure in rabbits alters gut microbiota and their metabolite profiles, which leads to colonic and liver inflammation as well as to increased gut permeability as measured by elevated serum lipopolysaccharide (LPS) levels in the offspring. Also, perinatal BPA exposure leads to reduced levels of gut bacterial diversity and bacterial metabolites (short-chain fatty acids [SCFA]) and elevated gut permeability—three common early biomarkers of inflammation-promoted chronic diseases. In addition, we showed that SCFA ameliorated BPA-induced intestinal permeability *in vitro*. Thus, our study results suggest that correcting environmental toxicant-induced bacterial dysbiosis early in life may reduce the risk of chronic diseases later in life.

## INTRODUCTION

Environmental factors present during early development may influence disease susceptibility later in life (1). The hypothesis of “fetal origins of adult disease” suggests that nutritional or chemical influences in early life have lasting effects (2). Bisphenol A (BPA), an environmental endocrine disruptor, is one of the most prevalent chemical estrogens in consumer products in daily use. BPA is widely used in plastics and in the epoxy resins which line food and beverage cans. The estimated annual global production of BPA is approximately 6 million tons (3) and is expected to rise in coming years. BPA can transfer across the placenta (4), and the presence of BPA in umbilical cord blood, amniotic fluid, fetal tissues, and breast milk reveals the possibility of significant early exposure to BPA (4–6). Evidence from animal studies indicates that BPA exposure at doses well below the tolerable daily intake (TDI) of 50 μg/kg of body weight/day established by the U.S. Environmental Protection Agency and the European Food Safety Agency is associated with cancers and reproductive and behavioral abnormalities (7, 8). Other studies have indicated that chronic exposure to BPA at the TDI or at the no-observed-adverse-effect-level (NOAEL) dose of 5 mg/kg/day caused alterations in reproductive development, hormone secretion, endocrine function, and childhood behavior (9–11). BPA is hormonally active at low concentrations, and the persistence of unconjugated BPA levels in plasma indicates chronic BPA exposure to target tissues such as colon and liver.

BPA exposure is associated with liver health as BPA is primarily metabolized by the liver (12). Moreover, the accumulation of BPA was observed in fetal liver and gut, with a relatively high concentration seen in the fetal gut upon perinatal exposure (13). However, relatively few studies have focused on the effect of BPA exposure on intestinal health. BPA exposure showed a positive association with proinflammatory responses in the colon of female but not male offspring (14). Variation in the composition of the microbiome could underlie these differences in association based on gender. Gut bacteria play a critical role in both colonic and liver inflammation. BPA exposure has been shown to alter the gut bacterial composition (15, 16) and metabolite profiles (17, 18) in mice. Gut bacterial dysbiosis is associated with increased intestinal permeability and pathogenesis of intestinal disorders (19). Moreover, alterations in host gut metabolites and bacterial endotoxins such as lipopolysaccharide (LPS) and increased paracellular translocation of gut-derived elements into the portal vein may activate proinflammatory gene expression and promote the inflammatory response in the liver and the onset of diet-induced hepatic steatosis and insulin resistance (20). Therefore, we hypothesized that perinatal exposure to BPA induces colon and liver inflammation via alterations in gut bacterial composition and metabolite profiles.

Accordingly, we aimed to determine the impact of perinatal BPA exposure on colonic and liver inflammation, colonic permeability, and the gut microbiome and colonic and liver metabolome of rabbits. To perform the analyses, we utilized tissue samples from a developmental and reproductive toxicology study testing the acute toxicity of BPA. This study used rabbits as an animal model, because this species has a long infantile period of development similar to that of humans and unlike that of rodents (21) and utilized BPA at a relatively low dose level of 200 μg/kg body weight/day (approximately 1/25 of the NOAEL dose). Although it would have been ideal to have a range of doses tested, it was not feasible because of the limited resources for this pilot study.

First, we examined the inflammatory response in the colon and liver of male offspring at 6 weeks of age through determination of levels of lymphocyte and neutrophil infiltration. Second, since BPA increased the inflammatory response in both colon and liver, which may have been due to gut bacterial dysbiosis, we evaluated the effects of perinatal BPA exposure on fecal microbiome and colon and liver metabolome. Significant alterations were observed in the microbiome and metabolome, including microbial metabolites such as short-chain fatty acids (SCFA). We observed increased gut permeability as measured by elevated systemic LPS levels upon BPA exposure, which may also contribute to the increased liver inflammation. Finally, we tested the hypothesis that supplementation of SCFA may ameliorate BPA-induced increases in colonic cell permeability *in vitro*.

## RESULTS

To evaluate the effects of perinatal BPA exposure on the offspring colon and liver, pregnant rabbits received 200 µg of BPA/kg of body weight/day orally from gestation day 15 through postnatal day 7. BPA exposure did not significantly influence litter size, survival, birth weight, or sex ratio (see Table S1 in the supplemental material).

10.1128/mSystems.00093-17.3 TABLE S1  Effect of BPA on litter size and sex ratio. Download TABLE S1, PDF file, 0.01 MB. Copyright © 2017 Reddivari et al. 2017 Reddivari et al. This content is distributed under the terms of the Creative Commons Attribution 4.0 International license.

### Effects of BPA exposure on the inflammatory response in the rabbit offspring.

Degenerative changes in hepatocytes and inflammatory cell infiltration in the lamina propria of the colonic mucosa and periportal region of hepatic lobules were observed at various levels of severity 6 weeks after cessation of the BPA treatment. Inflammatory cell infiltration in the colonic lamina propria and hepatic periportal region were more frequent in BPA-treated dams than in the controls (2 dams versus 1 and 3 versus 1, respectively) as well as in 6-week-old BPA-exposed rabbit offspring (5 pups versus 0 and 6 versus 1) (Table 1). The total numbers of pups with scores of grades 1 to 3 (0 = no lesions; 3 = severe lesions) for inflammatory cells in the colonic lamina propria and in the hepatic periportal region were significantly higher (Fisher exact test; *P* < 0.01) in BPA-treated animals than in controls.

**TABLE 1:** Incidence of lesions in liver and colon

Animal and lesion parameter	Value^a^					
	Colon		Liver			
	Inflammatory cells—lamina propria	Lymphocytes— laminapropria	Hydropic degeneration	Fat deposition	Lymphocyte infiltration	Inflammatory cells—periportal region
Control pups						
Grade 0	10	8	3	6	8	9
Grade 1		2	4	1	2	1
Grade 2			2	2		
Grade 3			1	1		
Grades 1–3	0^b^	2	7	4	2	1^b^
						
BPA pups						
Grade 0	2	3	3	8	8	2
Grade 1	5	4	1	0		3
Grade 2			3	0		3
Grade 3			1	0		
Grades 1–3	5^b^	4	5	0	0	6^b^

^a^ H&E-stained sections were scored based on a grade of 0 to 3 (0 = no lesions; 3 = severe lesions).

^b^ Data were significant at a *P* value of <0.05 (Fisher exact test).

**FIG 1:**
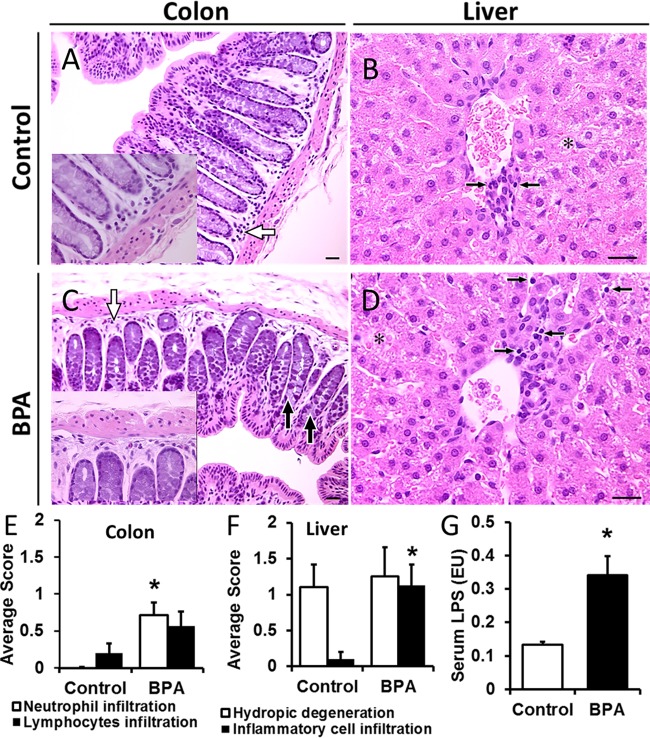
The effect of bisphenol A (BPA) on colon and liver inflammation. Rabbit offspring were exposed *in utero* or through lactation to either vehicle or BPA (200 μg/kg/day) from gestation day 15 through postnatal day 7, and tissues were collected at 6 weeks of age. (A to D) Representative images of colon and liver sections stained with H&E (scale bars = 25 µm). (A) Normal colonic mucosa in a control pup. The white arrow indicates a normal distribution of lymphocytes (inset: high magnification). (B) Liver parenchyma in a control pup. Lymphocytic infiltration (black arrows) was sparse, which is typical of a normal liver. The asterisk indicates areas of hydropic degeneration with possible accumulation of glycogen in hepatocytes, which was also observed in BPA-exposed offspring (see panel D). (C) Colonic inflammation in a BPA-exposed pup. Black arrows indicate coagulative necrotic changes in mucosal epithelium; the white arrow indicates focal neutrophil and eosinophil infiltration in the lamina propria (inset: high magnification). (D) Liver parenchyma in a BPA-exposed pup showing moderate (grade 2) infiltration of inflammatory cells. Black arrows point to some of the lymphocytes seen in the micrograph. Note that the cytoplasmic features of patches of hepatocytes (asterisk) in this BPA-exposed pup are similar to those seen in the control pup (shown in panel B). (E) Comparison of magnitudes of lesions in the colon. (F) Comparison of magnitudes of lesions in the liver. H&E-stained sections were scored based on a scale of 0 to 3 (0 = no lesions; 3 = severe lesions). (G) Serum lipopolysaccharide (LPS) levels were high in BPA-exposed offspring compared to controls. LPS was measured by the Endochrome-K Kinetic Chromogenic LAL assay. Data are expressed as means ± standard errors of the means (SEM) (*n* = 9 or 10 rabbit pups per group). The Wilcoxon rank sum test was used to calculate statistical significance. *, *P* < 0.05 (versus controls).

Examples of hydropic degenerative changes and periportal inflammatory cell infiltration are shown in Fig. 1. Isolated degenerative changes in colonic enterocytes were also seen. The severity of lesions in hematoxylin and eosin (H&E)-stained sections was scored based on the scale of 0 to 3. Average scores for inflammatory cell infiltration were increased significantly with BPA exposure (Fig. 1E and F). Chronic colonic inflammation can cause an alteration in epithelial barrier function leading to increased levels of systemic lipopolysaccharide (LPS). Systemic LPS levels were significantly higher in BPA-exposed offspring than in the controls (Fig. 1G), suggesting increased gut permeability. Interestingly, when a BPA-treated dam and pup were examined at day 1 to day 2 postpartum, the lesions were more intensive and the infiltration of inflammatory cells in both the liver and colon was striking in the neonate (see Fig. S1 in the supplemental material).

10.1128/mSystems.00093-17.1 FIG S1  The effect of bisphenol A (BPA) on liver and colon inflammation of neonates. BPA-treated dam and a pup exposed to BPA (200 μg/kg/day) *in utero* from gestation day 15 to birth were examined 1 to 2 days postpartum. (A to D) Representative images of liver and colon sections stained with hematoxylin and eosin (H&E) (scale bars = 25 µm). (A) Colonic inflammation and deformation of crypt architecture in a BPA-exposed neonate. (B) Liver parenchyma in a BPA-exposed neonate showing infiltration of inflammatory cells. (C) Normal colonic mucosa with limited inflammatory cell infiltration in a BPA-treated dam. (D) Hepatic steatosis in BPA-treated dam. During tissue preparation, the lipid dissolved, leaving white spaces where the fat would have been stored as macrovesicular deposits. The infiltration of inflammatory cells in both the liver and colon were striking and the lesions were more intensive in the neonate shown in panel A and B. Download FIG S1, TIF file, 1.2 MB. Copyright © 2017 Reddivari et al. 2017 Reddivari et al. This content is distributed under the terms of the Creative Commons Attribution 4.0 International license.

### Effect of perinatal BPA exposure on the microbiome.

**FIG 2:**
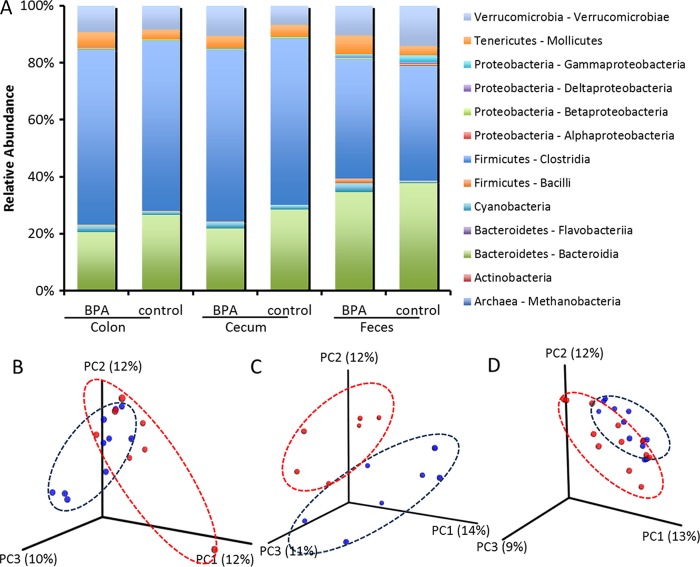
Microbial responses to the perinatal bisphenol A (BPA) exposure in rabbit offspring. (A) Relative abundances of major taxonomic classes in colon, cecum, and feces of rabbit offspring at 6 weeks of age where pregnant does were dosed orally with 0 or 200 μg/kg/day BPA from gestation day 15 through postnatal day 7. Taxonomy plots were generated by grouping samples by body site and BPA treatment. Samples with fewer than 2,000 sequences per sample were filtered out, as were rare taxa (minimum of 0.1% abundance in at least one category and present in at least 10% of samples). (B to D) Beta diversity plots for colon (B), cecum (C), and feces (D) with samples clustering according to the unweighted UniFrac metric.

Colon, cecum, and fecal microbiotas of rabbit dams and offspring were profiled using 16S rRNA gene sequencing (*n* = 9 to 11 rabbits/treatment). Phylogenetic classification of operational taxonomic units (OTUs) showed two predominant phyla, *Bacteroidetes* and *Firmicutes*, for both treatment groups in colon, cecum, and feces. The relative levels of abundance of different classes of bacteria are shown in Fig. 2A.

We observed distinct clustering by treatment group in considering each body site independently (Fig. 2B to D). Beta diversity levels were calculated using unweighted UniFrac (22) distances and were significantly different between BPA-exposed offspring and control offspring for cecum and feces (permutational multivariate analysis of variance [PERMANOVA] of unweighted UniFrac distances; *P* values were 0.12, 0.001, and 0.01 for colon, cecum, and feces, respectively). However, PERMANOVA of weighted UniFrac distances did not show any significance in the results of comparisons between BPA-exposed and control offspring (*P* values were 0.136, 0.68, and 0.067 for colon, cecum, and feces, respectively). Measures of alpha diversity, including Chao1, Simpson, and phylogenetic diversity (PD) indices, were similar in the two groups for colon, cecum, and feces.

**FIG 3:**
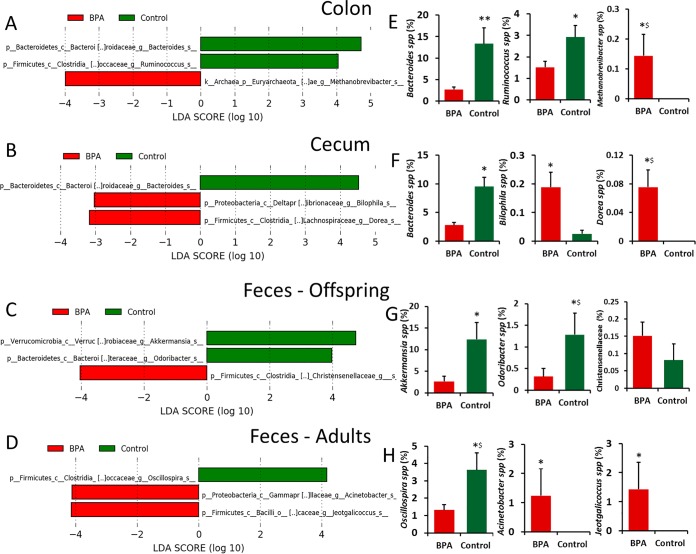
Differences in microbiome between control and BPA-exposed rabbit offspring and dams according to linear discriminant analysis effect size (LEfSe). Publicly available LEfSe visualization tools were used to generate these graphical outputs using BPA and control as class variables for colon, cecum, and fecal samples from offspring and fecal samples from dams separately. (A to D) Histogram of the linear discriminant analysis (LDA) scores calculated for features that were differentially abundant between BPA-treated and control rabbits. LDA scores that were elevated in control dams or offspring are shown in green, whereas LDA scores that were greater in BPA-treated dams or offspring are shown in red. Significant differences were identified by the use of the LEfSe algorithm with *P* < 0.05 for both the Kruskal-Wallis test and Wilcoxon test. (E to H) Relative abundances (%) of taxa that were significantly different between control and BPA treatments using the LEfSe algorithm. *, significance of results of comparisons between treatments at *P* ≤ 0.05 using the Wilcoxon rank sum test. **, significance at *P* ≤ 0.05 using Bonferroni correction. $, significance at *P* ≤ 0.1 using Bonferroni correction.

To study the differences in fecal microbiota between BPA-exposed and control dams and offspring, the linear discriminant analysis effect size (LEfSe) algorithm was used for determinations of bacterial abundance (Fig. 3). We observed an increase in the relative abundance of *Methanobrevibacter* spp. in the colon of male offspring exposed to BPA. The relative levels of abundance of *Bacteroides* spp. and *Ruminococcus* spp. were higher in the colonic digesta of control offspring than in that of BPA-treated offspring (Fig. 3A and E). With respect to the cecum, the relative abundance of *Dorea* spp. and *Bilophila* spp. was increased and that of *Bacteroides* spp. was reduced in BPA-exposed male offspring (Fig. 3B and F). We observed significant positive correlations between the abundance of colon *Methanobrevibacter* spp. and systemic LPS levels (*r*^2^ = 0.67; *P* = 0.023) and between the abundance of cecal *Dorea* spp. and systemic LPS levels (*r*^2^ = 0.82; *P* = 0.004). The abundances of *Ruminococcaceae* and *Coprococcus* spp. were negatively correlated with systemic LPS levels (*r*^2^ = −0.72 and −0.63; *P* = 0.013 and 0.05, respectively).

Fecal samples were analyzed for both adults and offspring. In control offspring, the relative abundance of *Akkermansia* spp. and *Odoribacter* spp. was greater than the relative abundance seen under conditions of exposure to BPA (Fig. 3C and G). The relative abundance of *Oscillospira* spp. was higher in control dams than in BPA-administered dams. The abundance of *Acinetobacter* and *Jeotgalicoccus* was higher among fecal microbiota of the BPA-administered dams than among fecal microbiota of control dams (Fig. 3D and H).

The abundance of *Oscillospira* spp., which represented at least 1% of the bacterial population in both treatments, showed significance (*P* = 0.08 [Fisher’s least significant difference {LSD} after correcting for multiple comparisons]) in comparisons between control and BPA-administered dams. The relative levels of abundance of *Odoribacter* spp. were significantly (*P* = 0.1) different in comparisons between control and BPA-exposed offspring. The abundance of *Akkermansia* spp. was found to have dropped from 12% to 2.6% in offspring upon BPA exposure (Fig. 3G). This suggests that the observed differences in the levels of gut bacteria in the BPA pups may have been partly due to differences due to BPA exposure in the dams and partly due to the effect of BPA *in utero* on the pup sterile gut environment and (postnatally) on gut microbiota development.

### Effect of BPA exposure on SCFA and cell permeability.

**FIG 4:**
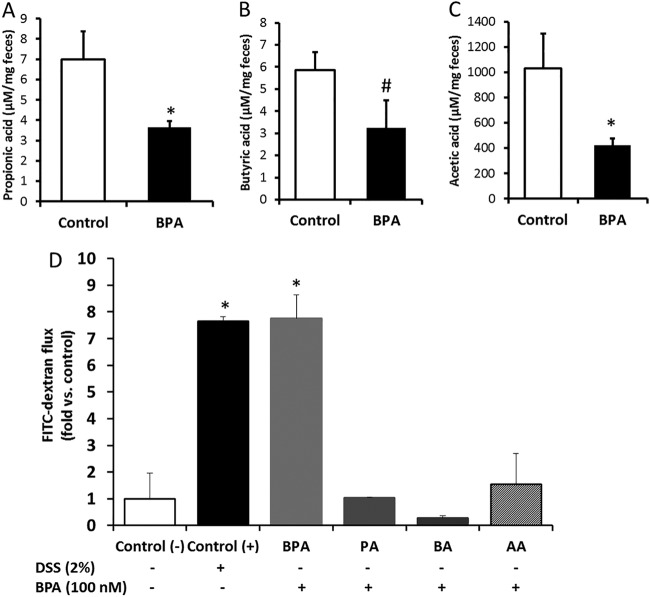
(A to C) Fecal short-chain fatty acid levels (based on dry weights) in the offspring exposed to vehicle and BPA. Data are expressed as means ± SEM (*n* = 8). *, significant difference from control at *P* ≤ 0.05; #, significant trend in comparisons between control and BPA at *P* ≤ 0.1. (D) Cell permeability analysis using apical to basolateral flux of fluorescein isothiocyanate-dextran (FITC-dextran) in Caco-2 cells exposed for 24 h to vehicle [control (-)] or dextran sodium sulfate (DSS; 2%) or BPA (100 nM) with or without propionic acid (PA) or butyric acid (BA) and acetic acid (AA). Data are expressed as means ± SEM (*n* = 4) of results from duplicate experiments. *, significant difference from the negative control at *P* < 0.05.

Alterations in gut microflora and inflammatory changes can influence SCFA levels and enterocyte permeability (23). Levels of colonic gut bacterial metabolites acetic acid and propionic acid were significantly reduced in the feces of BPA-exposed offspring compared to controls. In BPA-exposed offspring, there was a significant trend toward a reduction in butyric acid levels (Fig. 4A to C). To investigate whether BPA affects cell permeability *in vitro*, human colonic Caco-2 cells were used. Caco-2 monolayers were treated either with BPA (50 and 100 nM) or vehicle (ethanol). BPA treatment caused a dose-dependent increase of colonic cell permeability as measured by apical-to-basolateral flux of fluorescein isothiocyanate-dextran (FITC-D). The FITC-D transfer rate increased from 9.8 mg/h in the control cells to 54.2 mg/h in the BPA (100 nM)-treated cells. To determine whether the increase in colonic cell permeability was related to alterations in bacterial metabolites, BPA-treated cells were supplemented with acetate, propionate, or butyrate in ratios similar to those observed in human feces (48 mM, 16 mM, and 16 mM). SCFA supplementation restored the BPA-induced increase in cell permeability to levels similar to those of control cells (Fig. 4D). 

### Effects of BPA exposure on the host metabolome. (i) Untargeted analysis.

**FIG 5:**
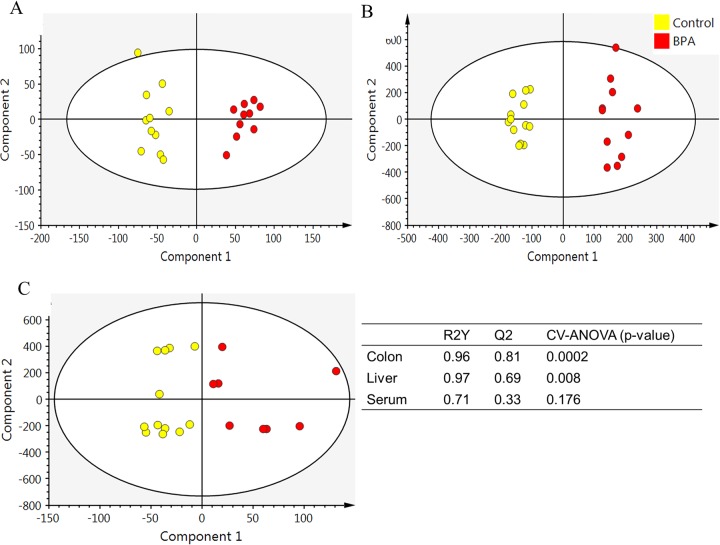
Orthogonal partial least-squares discriminant analysis (OPLS-DA) plot for LC-MS/MS metabolite features. Rabbit offspring were exposed to either vehicle or BPA for 15 days during gestation, and liver, colon mucosa, and serum samples were collected at 6 weeks of age. Data represent OPLS-DA results for (A) colon metabolites (R2Y = 0.96 and Q2 = 0.81), (B) liver metabolites (R2Y = 0.97 and Q2 = 0.69), and (C) serum metabolites (R2Y = 0.71 and Q2 = 0.33) of control rabbit pups (*n* = 13) and BPA rabbit pups (*n* = 9 to 11). R2Y and Q2 values should be higher than 0.5 for good OPLS-DA models.

Global metabolite profiles of liver, colon mucosa, and serum were assessed in both control and BPA-exposed offspring. Orthogonal partial least-squares discriminant analysis (OPLS-DA) of comparisons of the control group to the BPA group generated an OPLS-DA model with latent parameters, characterized by the goodness of the fit of the data (R2Y) and cumulative predictive capacity (Q2). Distinct clustering of colon, liver, and serum metabolite features between control and BPA-exposed offspring was observed (Fig. 5). R2Y values of 0.96, 0.97, and 0.71 for colon, liver, and serum, respectively, indicate accurate representation of the data. Q2 values of 0.81 and 0.69 for colon and liver represent great cumulative predictive capacity. Q2 values for serum were low (Fig. 5). The score plots of the OPLS-DA data for colon, liver, and serum showed a clear separation between the BPA group and the control group. Approximately 550 metabolite features had a variable importance in projection (VIP) value of >2.0, and 100 features which were not annotated were significantly different between the control group and the BPA group with respect to liver data after false-discovery-rate (FDR) correction. Only three colon metabolite features showed a trend toward significance (*P* ≤ 0.1) in the comparisons between the control and BPA groups. None of the results of comparisons of serum metabolite features were significant after FDR correction.

### (ii) Targeted analysis.

**FIG 6:**
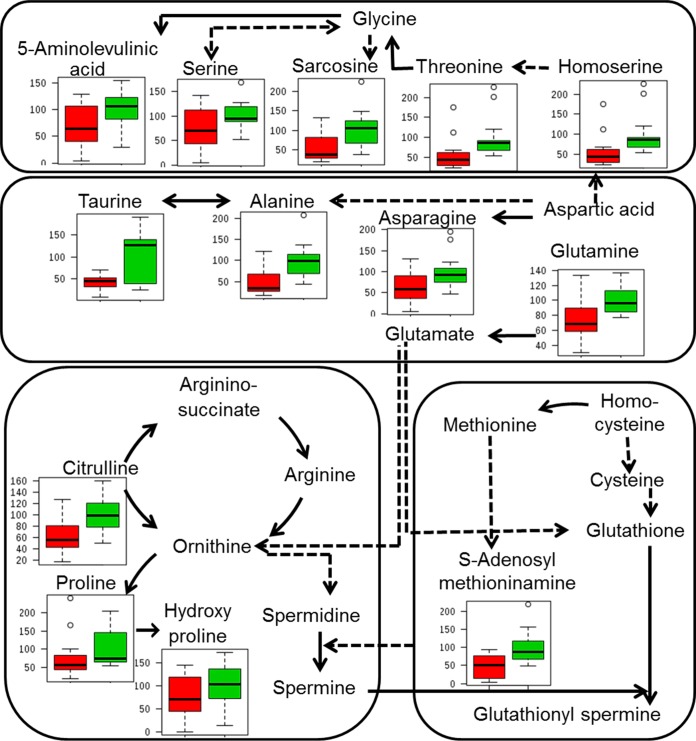
Effect of BPA exposure on liver amino acid metabolism. Solid lines represent direct conversion. Dotted lines represent intermediates. Box plots show the percentages of relative intensity levels compared to control results. Green bars represent control offspring, and red bars represent BPA-exposed offspring.

Gut bacterial dysbiosis has been shown to alter amino acid metabolism. Global metabolite profiles of colon and liver generated in negative mode were analyzed for targeted amino acids and simple sugars using an in-house database of standards. In the colon, only histidine levels were significantly reduced in the control offspring compared to the BPA-exposed offspring. The liver metabolites whose levels were significantly different after adjusting for multiple testing (FDR) were reduced in BPA-exposed offspring compared to control offspring (Table S2). Amino acids and their metabolites were significantly altered in glycine-serine-threonine metabolism, alanine-aspartate-glutamate metabolism, arginine-proline metabolism, and cysteine-methionine metabolism in BPA-exposed offspring compared to control offspring. Levels of homoserine, threonine, alanine, glutamine, citrulline, and S-adenosyl-l-methioninamine (SAM) were significantly reduced in BPA-exposed offspring (Fig. 6). Ribulose-5-phosphate, dihydroxy-acetone-phosphate, and d-glyceraldehdye-3-phosphate levels showed a 2-fold difference between control offspring and BPA-exposed offspring. Values corresponding to the area under the curve, normalized peak intensity, sensitivity, specificity, and maximum Youden index for each significant metabolite as calculated by receiver operating characteristic (ROC) curve analysis are shown in Table S3. Pathway analysis revealed that histidine metabolism and beta-alanine metabolism in the colon were significantly altered upon BPA exposure. In liver, several pathways were significantly altered upon BPA exposure, with taurine-hypotaurine metabolism, alanine-aspartate-glutamate metabolism, and arginine-proline metabolism having a high impact factor (Fig. S2; Table S4). A high pathway impact factor indicates that the pathway is greatly influenced.

10.1128/mSystems.00093-17.2 FIG S2  Pathway analysis of targeted metabolites of the colon and liver showing the significance and the impact. The pathways that are significantly different between BPA and control offspring are listed in Table S4. Download FIG S2, TIF file, 1.3 MB. Copyright © 2017 Reddivari et al. 2017 Reddivari et al. This content is distributed under the terms of the Creative Commons Attribution 4.0 International license.

10.1128/mSystems.00093-17.4 TABLE S2  Metabolites identified as differentially expressed between control and BPA-exposed offspring. Download TABLE S2, PDF file, 0.02 MB. Copyright © 2017 Reddivari et al. 2017 Reddivari et al. This content is distributed under the terms of the Creative Commons Attribution 4.0 International license.

10.1128/mSystems.00093-17.5 TABLE S3  Receiver operating characteristic curve analysis of significant metabolites—targeted analysis. Download TABLE S3, PDF file, 0.1 MB. Copyright © 2017 Reddivari et al. 2017 Reddivari et al. This content is distributed under the terms of the Creative Commons Attribution 4.0 International license.

10.1128/mSystems.00093-17.6 TABLE S4  Pathways that were significantly different between BPA-exposed and control offspring. Download TABLE S4, PDF file, 0.02 MB. Copyright © 2017 Reddivari et al. 2017 Reddivari et al. This content is distributed under the terms of the Creative Commons Attribution 4.0 International license.

## DISCUSSION

Here we report that perinatal BPA (200 µg/kg of body weight/day) exposure-induced colonic and liver inflammation is associated with an altered composition of the metabolites and of microbiota in the male offspring of rabbits. We focused on the colon and liver, as BPA accumulation was observed in the fetal gut and liver, with a relatively high concentration in the fetal gut (13). Furthermore, BPA is mainly metabolized in human liver (24). We showed that BPA exposure increased the inflammatory cell infiltration in the colon of rabbit offspring (Fig. 1). Similarly, exposure to environmental toxins, including BPA, has been shown to be associated with an increased incidence of colonic inflammation, neutrophil infiltration, and colon cancer (25, 26). BPA exposure caused inflammatory cell infiltration in the periportal region of hepatic lobules (Fig. 1; Table 1) that is associated with hepatitis (27). This may have been due to the reduction in the level of liver UDP-glucuronosyltransferase that metabolizes BPA leading to active BPA accumulation sufficient to cause cytotoxicity of hepatocytes (28, 29). The increase in serum endotoxin levels was observed in patients with either liver or colon diseases (30, 31). Accordingly, we show that the liver and colonic inflammation induced by BPA exposure was associated with elevated systemic LPS levels in male rabbit offspring (Fig. 1G), indicating increased gut permeability. Increased levels of systemic LPS can translocate into the liver via the portal vein (20) and activate proinflammatory gene expression followed by cytokine production, which in turn can promote the onset of diet-induced hepatic steatosis (32). We observed the inflammation in the offspring exposed to BPA and hepatic steatosis in BPA-consuming dams (Fig. S1) (33), suggesting that offspring are at greater risk of developing hepatic steatosis later in life. Here we show that alterations in the bacterial composition of the gut represent one possible explanation for BPA-induced liver and colonic inflammation and increased systemic LPS levels. Gut bacterial dysbiosis may alter the bacterial metabolites and may weaken the tight junctions, allowing paracellular translocation of lymphocytes and endotoxins into the portal vein and liver. These bacterial endotoxins may increase inflammation and oxidative stress in the liver through activation of Toll-like receptors (TLRs) (20).

Significant differences between BPA-exposed and control rabbits in colon and cecum microbiome composition and a significant trend in the results seen for the fecal microbiome were observed (Fig. 2). A recent study (16) reported significant differences in fecal microbial composition between offspring of unexposed mice and offspring with parental exposure to BPA. In CD-1 mice, BPA intake changed gut microbial composition in ways similar to those seen with mice consuming high-fat or high-sucrose diets that are implicated in several inflammation-promoted chronic diseases (15). We observed differences between BPA and control male offspring in the genera *Bacteroides* (*Bacteroidaceae*), *Methanobrevibacter*, and *Ruminococcus* (*Ruminococcaceae*) in the colon and in the genera *Bacteroides*, *Dorea* (*Lachnospiraceae*), and *Bilophila* in cecum. However, after correcting for multiple comparisons, only the results seen with *Bacteroides* spp. in the colon were statistically significant. The genera *Methanobrevibacter* in the colon and *Dorea* in the cecum showed significance at *P* values of ≤0.1 after Bonferroni correction in comparisons between BPA-exposed offspring and control offspring (Fig. 3). *Bacteroides* spp. play an important role in immune modulation and inflammation. *Methanobrevibacter* levels were increased in mothers exposed to BPA. These *Archaea* can metabolize dietary substrates that lead to increased host energy intake and weight gain (34). *Ruminococcaceae* and *Lachnospiraceae* taxa were correlated with protection against *Clostridium difficile* infection (35). *Ruminococcaceae* was also shown to be associated with lower long-term weight gain and improved energy metabolism in mice (36).

In fecal samples, a significant decrease in the relative abundance of *Oscillospira* spp., butyrate producers in the family of *Ruminococcaceae*, was observed in BPA-exposed dams compared to controls. Levels of *Oscillospira* spp. were also significantly reduced in several cases of diseases that involve inflammation (37). In the male offspring exposed to BPA, levels of *Akkermansia* spp. and *Odoribacter* spp. were decreased compared to the control group (Fig. 3). However, *Bacteroides*, *Odoribacter*, and *Akkermansia* levels were elevated in tumor-bearing mice (38). Levels of *Odoribacter*, a butyrate-producing bacterium, and *Akkermansia* are inversely correlated with systolic blood pressure and colonic inflammation, respectively (39). *Akkermansia* spp. have also been proposed as a target for probiotic treatment (40), suggesting that BPA-induced inflammation may be due to the negative effects of BPA exposure on beneficial microbes. BPA-induced inflammation was associated with changes in gut bacterial composition; however, whether gut bacterial dysbiosis is truly a cause or merely a consequence of inflammation is still debatable. Although evidence from experimental models indicates a consistent role of gut bacteria in chronic inflammation, the precise role of microbial dysbiosis is less clear (41). BPA-induced inflammation was associated with changes in gut bacterial composition; however, whether these changes were a cause or a consequence of inflammation is still debatable.

Some of the bacteria (*Ruminococcaceae*, *Oscillospira* spp., and *Odoribacter* spp.) that are significantly altered by BPA exposure are important producers of SCFA (42). Accordingly, we observed a significant reduction in acetic acid and propionic acid levels and a trend toward reduction in butyric acid levels in rabbit offspring exposed to BPA (Fig. 4). The importance of SCFAs such as propionate and butyrate has been demonstrated in human studies. Propionic acid has been shown to be protective against carcinogenesis and colorectal cancer in humans (43). Butyrate increased the expression of tight junction proteins, thereby improving colonic defense barriers and protection against colitis, and the BPA-induced effect on colonocyte permeability may have been partly due to a reduction in butyrate levels (44–46). The reduction in the SCFA levels in BPA-exposed offspring may have been due to the reduced production and/or increased utilization owing to gut bacterial dysbiosis and gut permeability alterations.

Colitis and alcoholic liver disease patients showed increased intestinal permeability and altered fecal microbiota composition. In alcoholic liver disease patients, a drastic decrease in the abundance of bacteria belonging to the *Ruminococcaceae* family was observed. *Ruminococcaceae* was negatively correlated with intestinal permeability, while the genus *Dorea* was positively correlated with intestinal permeability (47). In line with this, we showed significant positive correlations between the abundances of colonic *Methanobrevibacter* spp. and cecal *Dorea* spp. and systemic LPS levels and negative correlations between cecal *Ruminococcaceae* and LPS levels. This suggests that the BPA-induced dysbiosis in the fecal microbiome might influence colonic cell permeability and colonic inflammation. However, BPA exposure in rats caused a reduction in colonic paracellular permeability instead of an increase in female offspring but not in male offspring. This may have been due to the overexpression of ERβ, which shows 10-fold-better affinity for BPA in females and a marked reduction in ERβ mRNA expression in males due to BPA exposure (14). In our study, we observed increased Caco-2 cell permeability *in vitro* upon BPA exposure at 100 nM (Fig. 4D). Though we did not measure the *in vivo* colon permeability directly, increased systemic LPS levels suggest increased cell permeability in BPA-exposed rabbit male offspring. This may have been due to the differential effects of BPA on cell permeability based on gender and concentration (14). Future studies are needed to understand species dependency on BPA-induced alterations in cell permeability. Interestingly, exposing the cells to SCFA alleviated the BPA-induced increase in cell permeability. These findings suggest the possibility of the use of SCFA-producing bacterial strains as probiotics against BPA exposure. Also, as some prebiotics are known to support the growth of SCFA-producing bacteria, prebiotics could be evaluated for their ability to prevent or reverse the BPA-induced inflammation in the colon and liver.

Toxicant-induced changes in gut microbiota could also alter the bacterial metabolites and key metabolic activities and thereby the host global metabolite profiles. The distinct clustering of metabolites seen using the OPLS-DA model was significant only for colon and liver metabolites (Fig. 5) and not for serum metabolites, indicating that the metabolome changes occurred initially in the tissues where the BPA accumulates. Additional studies are warranted to determine the effect at later stages and also the effect of long-term BPA exposure on systemic inflammation. Targeted analysis of global metabolome revealed significant alterations in levels of amino acids and simple sugars associated with inflammation and oxidative stress. Levels of S-adenosyl-l-methioninamine, a precursor molecule in the synthesis of glutathione that plays a critical role in natural defense against oxidative stress and inflammation, were significantly reduced with BPA exposure (48). Amino acids such as alanine and threonine that are significantly altered by BPA exposure are also involved in oxidative stress (49). Taurine-hypotaurine, alanine-aspartate-glutamate, and arginine-proline metabolism pathways were significantly altered between control and BPA-exposed offspring (Fig. 6; Tables S2 to S4). Moreover, these pathways are also involved in oxidative stress, inflammation, and immunity, confirming the role of BPA in inflammation. Taurine has been shown to protect tissues from oxidative stress associated with various inflammatory diseases (50). Glutamate and aspartate reduced levels of serum malondialdehyde and increased levels of anti-inflammatory factors in boars with hydrogen peroxide-induced oxidative stress (51). Proline has been shown to offset the cellular imbalances caused by environmental stresses (52). These results suggest that BPA-induced liver inflammation might be due to increased levels of oxidative stress and reduced levels of antioxidant and anti-inflammatory compounds.

### Conclusions.

Our data obtained using a rabbit model for developmental toxicology with an infantile period of development similar to that of humans suggest that perinatal BPA exposure may cause gut bacterial dysbiosis and altered gut metabolite profiles. Perinatal BPA exposure resulted in alterations in the gut bacterial composition and reductions in the levels of beneficial bacterial metabolites such as short-chain fatty acids. BPA exposure also elevated levels of chronic colonic and liver inflammation as well as systemic levels of harmful bacterial endotoxins such as LPS, a putative biomarker of proinflammation, in the male offspring. However, further studies are needed to establish whether BPA-induced gut bacterial dysbiosis is a cause or a consequence of chronic inflammation.

These results suggest that the rabbit model that we established could be used to assess the anti-inflammatory activity of prebiotics, probiotics, nutraceuticals, and drugs and to develop safe and affordable preventive strategies against adverse effects of environmental toxicants. This is particularly important as bisphenol S (BPS), a purportedly safe BPA alternative, has also been shown to have toxic effects in mammals (53, 54).

## MATERIALS AND METHODS

### Animals.

Age-matched, timed nulliparous pregnant Dutch-Belted rabbits were procured 12 days after mating (Myrtle’s Rabbitry, Thompson Station, TN) and acclimatized at the Colorado State University (CSU) animal facility for 3 days before starting dosing. Rabbits were housed in individual cages under conditions of a 12-h light/12-h dark cycle. They were fed a standard laboratory diet (Teklad rabbit diet; Harlan Laboratories, Indianapolis, IN) *ad libitum* with free access to water. Ten pregnant does were ranked based on body weight and randomly assigned to two treatment groups (*n* = 4) so that weights were distributed evenly across the two treatment groups. The diet was supplemented with organic grass hay to maintain optimum health. All procedures were performed according to the guidelines stipulated by CSU’s Institutional Animal Care and Use Committee.

### Dosing regimen.

Pregnant does were dosed orally with 0 or 200 µg/kg of body weight/day BPA in pureed organic carrot vehicle (Earth’s Best, Boulder, CO) from gestation day 15 (midgestation) through postnatal day 7 to ensure exposure of pups during important developmental phases *in utero* and via milk, collectively called perinatal (aka gestational and lactational) exposure. This regimen encompasses defined prenatal (latter half of gestation) and postnatal (first week of nursing) periods. Does were weighed twice a week, and the dosage was calculated. Kindling (delivery of rabbit pups) occurred on day 30 or 31 of gestation. The average litter size in this breed of rabbits ranges from 2 to 6, and the sex ratio is unpredictable (Table S1). BPA exposure did not significantly influence litter size, survival, birth weight, or sex ratio. Pups (in entire litters, which ranged from 3 to 6 pups) were housed in the same cage with the dam until 6 weeks of age; the dam typically nurses offspring for about 4 weeks. Fecal samples were collected at weekly intervals throughout the duration of the experiment for microbiome analysis. At 6 weeks of age, 10 pups representing four litters for a control group and 8 pups representing four litters for a BPA-treated group were euthanized intravenously (i.v.) (using 100 mg pentobarbital/kg) along with their dams. Blood serum, colon, and liver samples were collected and processed for metabolomics, gut bacterial sequencing, and histology.

### Histology.

Tissue samples were fixed in buffered formalin and embedded in paraffin. Sections were cut at 5-µm thickness, mounted on glass slides, and stained with hematoxylin and eosin (H&E). Stained sections were evaluated using a research-quality microscope interfaced with a computerized imaging system (BX61; Olympus Imaging, Melville, NY). Each section was examined for any signs of morphological changes (such as hydropic degeneration and fatty swelling) and/or inflammatory reactions (such as infiltration of lymphocytes and neutrophils) and scored using a “0 to 3” scheme (0, absence of any lesions; 1, mild [focal] lesions; 2, moderate lesions; 3, diffuse lesions).

### Serum LPS.

Serum samples were diluted 1:100 with Limulus amebocyte lysate (LAL) reagent water and incubated for 15 min at 70°C in pyrogen-free tubes. LPS levels were measured in duplicate by the Endochrome-K Kinetic Chromogenic LAL assay (Charles River, Inc., PA) using a standard curve generated with known concentrations of LPS in the range of 0.005 endotoxin units (EU)/ml to 50 EU/ml.

### Bacterial sequencing.

Genomic DNA was extracted using a Powersoil DNA extraction kit (Mo Bio, Inc., Carlsbad, CA) from distal gut digesta, cecal, and fecal samples. Hypervariable region 4 of the 16S ribosomal RNA gene was then amplified and sequenced using an Illumina MiSeq system (55). Data were processed with QIIME 1.7.0 (56) (development version 7b1e6e8f2975950b3f8b9ff9698db5ee58522c5b). Sequences were demultiplexed using default quality settings and were clustered using a closed-reference approach (discarding reads that failed to match the reference database at 97% identity) using the Greengenes (57) May 2013 database; these clustered reads acquired the taxonomies associated with the Greengenes reference database. The default parameters and UCLUST software (58), version 1.2.22q, were used for clustering. We chose to use one fecal sample per rabbit for our cross-sectional data analyses in order to avoid the inflation of *P* values because of samples that were not “independent observations,” which is an assumption of the statistical tests used in this study.

### Short-chain fatty acids.

Short-chain fatty acids (SCFA) were measured in the fecal samples using a gas chromatography-flame ionization detector (GC/FID) (59) with minor modifications. Fecal samples (100 mg) were homogenized in 1 ml of water–0.5% phosphoric acid in a bullet blender (Next Advance, NY) for 2 min. After centrifugation at 17,000 × *g* for 10 min, the supernatant was extracted with an equal volume of ethyl acetate. The internal standard heptanoic acid (30 mM) was added to the organic phase to correct injection variability between the samples. The analysis was done on an Agilent 6890 GC/FID linked to a Gerstel MPS2 autosampler. Data obtained using the GC/FID were compared to standard curves obtained using analytical standards of SCFA.

### Cell permeability.

Caco-2 cells were seeded at a density of 2 × 10^5^ cells/well in transwell inserts (pore size, 0.4 µm) and allowed to differentiate for 21 days. The differentiated cells were exposed to either BPA (100 nM) or BPA and SCFA (propionate and butyrate at 16 mM and acetate at 48 mM). Dextran sodium sulfate (DSS) at 2% served as a positive control. Transepithelial electrical resistance (TEER) readings were taken at 0, 3, 6, 12, 18, and 24 h using a Millicell ERS-2 system (Millipore Corp., MA). Permeability studies were conducted using apical-to-basolateral flux of fluorescein isothiocyanate-dextran (FITC-D). Permeability was determined by taking a 50-µl sample from the basolateral layer every 30 min for a total of 3 h and measuring the fluorescence intensity (λ excitation [ex] = 485 nm, λ emission [em] = 538 nm) using a CLARIOstar microplate reader (BMG Labtech, Cary, NC).

### Metabolomics.

Methanolic extracts of colon, liver, and serum samples were used for metabolite profiling. Global data acquisition was performed using time of flight and Orbitrap mass analyzers for untargeted and targeted metabolite analyses, respectively.

### Untargeted metabolite analysis.

Global metabolite profiling was performed using a Waters ultraperformance liquid chromatography/quadrupole orthogonal acceleration time of flight tandem micro-mass spectrometer (UPLC/Q-Tof Micro MS). Briefly, chromatographic separation was performed on a Waters Acquity UPLC C_8_ column (1.8 μm particle size; 1.0 by 100 mm) using a gradient from 5% acidified methanol to 95% acidified methanol at a column temperature of 50°C and a flow rate of 140 μl/min. Column eluent was infused into a Waters Q-Tof Micro MS fitted with an electrospray source, and data were collected in positive ion mode. MS data (including retention times, *m/z*, and ion intensities) in MassLynx software (Waters) were converted to cdf format using Databridge software. Raw peak areas were normalized to total ion signal in R and subjected to statistical analyses. Identification and alignment of peaks for metabolites that were significantly different between the groups were performed based on accurate mass, isotopic pattern, and tandem MS (MS/MS) information obtained from Massbank (http://massbank.eu), Metlin (http://metlin.scripps.edu), The Human Metabolome Database (http://www.hmdb.ca), and ChemSpider (http://www.chemspider.com) (60, 61).

### Targeted metabolite analysis.

Samples were analyzed by LC-MS using an electrospray ionization method (62) for amino acids and simple sugars. Samples were separated on a Phenomenex (Torrance, CA) Synergi Hydro-RP C_18_ column (100 by 2.1 mm; 2.5 µm particle size) using a water-methanol gradient with 10 mM tributylamine and 15 mM acetic acid added to the aqueous mobile phase. The HPLC column was maintained at 30°C with a flow rate of 200 µl/min. Solvent A was 3% aqueous methanol with 10 mM tributylamine and 15 mM acetic acid; solvent B was methanol. The gradient was 0 min at 0% solvent B followed by 5 min at 20% solvent B, 7.5 min at 20% solvent B, 13 min at 55% solvent B, 15.5 min at 95% B, 18.5 min at 95% B, 19 min at 0% solvent B, and 25 min at 0% solvent B. An Exactive Plus Orbitrap mass spectrometer controlled by Xcalibur 2.2 software (Thermo, Fisher Scientific, CA) was operated in negative ion mode at maximum resolution (140,000) and was used to scan from *m/z* 85 to *m/z* 1000. MAVEN software was used with our in-house database of approximately 300 standard metabolites (amino acids and simple sugars) for identification.

Quality control (QC) samples were prepared by pooling equal aliquots of all the samples and were run at the beginning and end of the randomized sample set and after every 10 injections to assess the drift in retention time and variation in ion intensities. Metabolite features with a 0.3-min drift in retention time in QC samples were removed. A standard metabolite mix was also run at similar intervals along with QC samples to evaluate the performance of the instrument with respect to mass accuracy and sensitivity.

### Statistics.

Fisher's exact test was used for comparing the severities of lesions between the groups. The Wilcoxon rank sum test was used for comparing the histological scores. Fisher’s LSD and Bonferroni correction were used for determining significant differences between the groups for gut bacteria. Rarefaction metrics and β-diversity measurements were calculated in QIIME. LEfSe results were visualized using bar charts and cladogram plots produced using the Huttenhower laboratory Galaxy server (https://huttenhower.sph.harvard.edu/galaxy/). Spearman correlations of comparisons between bacterial abundance and systemic LPS levels were calculated using SPSS, Inc. Simca software was used to create OPLS-DA plots. Cross-validated ANOVA (CV-ANOVA) was used to test the significance of OPLS-DA models. Receiver operating characteristic (ROC) curves were used to identify the metabolites. MetaboAnalyst was used for pathway analysis and ROC curve analysis of metabolite features. For metabolites and gut bacteria, an adjusted *P* value of ≤0.05 after correcting for multiple comparisons using FDR or Bonferroni correction was considered significant.

### Data availability.

All sequences have been archived in the Qiita database under study identifier (ID) 839 (https://qiita.ucsd.edu/study/description/839) and archived in the European Nucleotide Archive of the European Bioinformatics Institute (EMBL-EBI) database under accession number ERP104394.
