# Supplementary material for: Perinatal Bisphenol A Exposure Induces Chronic Inflammation in Rabbit Offspring via Modulation of Gut Bacteria and Their Metabolites
Source: mSystems. 2017 Oct 10;2(5):e00093-17. doi: 10.1128/mSystems.00093-17 (PMC5634791; doi:10.1128/mSystems.00093-17)
Supplement: TABLE S1 [file sys005172142st3.pdf]

Supplementary Tables

Table S1: Effect of BPA on litter size and sex ratio

Dam	Treatment group	Total litter size	stillborn	Live @ post-natal day2	Males/ litter	Females/ litter
485	control	6	1	1	0	4
486	control	6	2	4	4	2
487	control	8	0	9	7	1
488	control	10	0	10	4	6
489	control	5	2	3	1	2
490	BPA	2	1	1	1	0
491	BPA	5	0	0	1	3
492	BPA	9	0	9	5	4
493	BPA	6	4	2	3	3
494	BPA	8	1	7	6	2
