# Supplementary figures and images for: Perinatal Bisphenol A Exposure Induces Chronic Inflammation in Rabbit Offspring via Modulation of Gut Bacteria and Their Metabolites
Source: mSystems. 2017 Oct 10;2(5):e00093-17. doi: 10.1128/mSystems.00093-17 (PMC5634791; doi:10.1128/mSystems.00093-17)

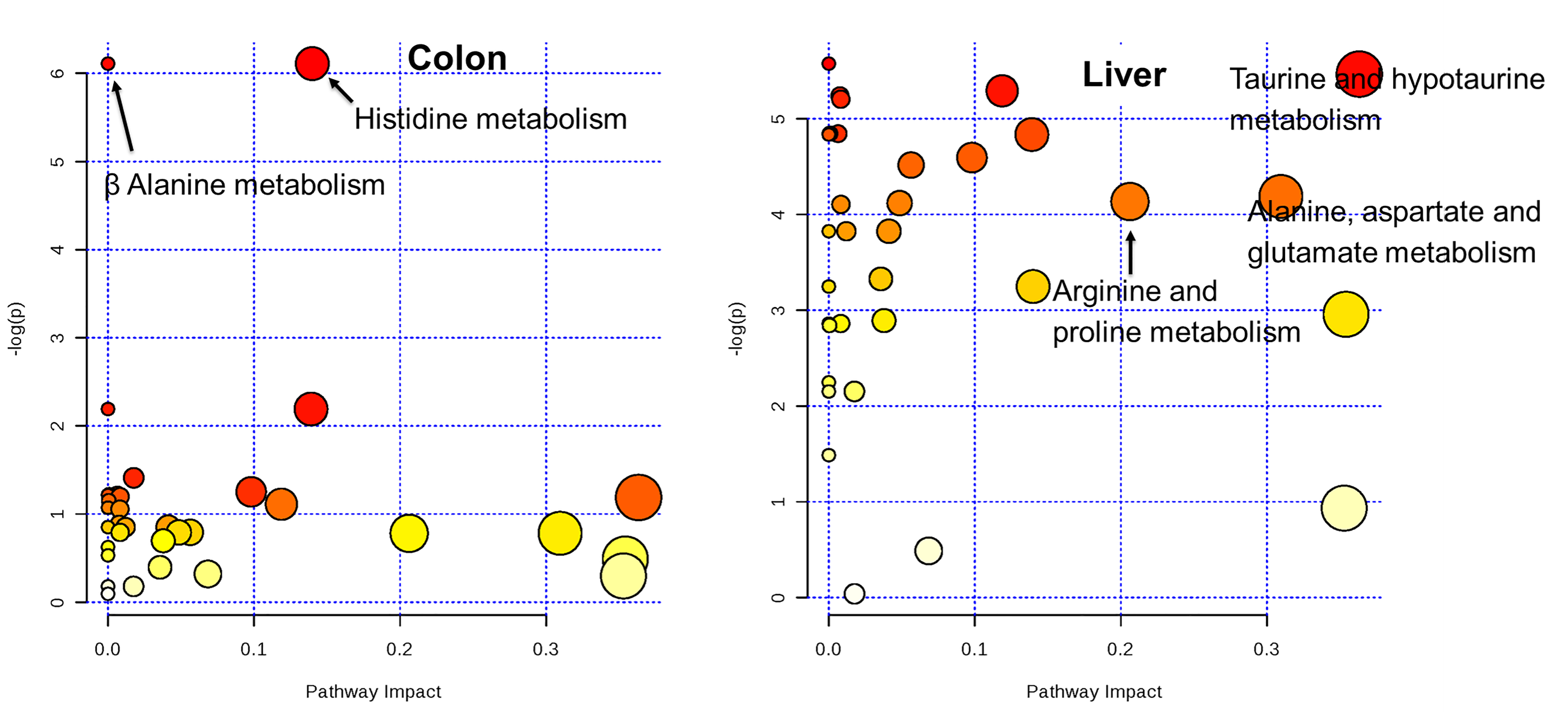

Supplement: FIG S2 [file sys005172142sf2.tif]
