# Supplementary material for: Perinatal Bisphenol A Exposure Induces Chronic Inflammation in Rabbit Offspring via Modulation of Gut Bacteria and Their Metabolites
Source: mSystems. 2017 Oct 10;2(5):e00093-17. doi: 10.1128/mSystems.00093-17 (PMC5634791; doi:10.1128/mSystems.00093-17)
Supplement: TABLE S2 [file sys005172142st4.pdf]

Table S2: Metabolites identified as differentially expressed between control and BPA-exposed offspring

RT (min)	Measured Mass, Da	Calculated Mass, Da	Elemental composition	Postulated identity	Fold change	VIP	Adj p-value
			**COLON**				
1.25	155.066	155.069	$C_6H_9N_3O_2$	Histidine	0.33	1.28	0.047
			**LIVER**				
1.25	355.152	355.155	$C_{14}H_{23}N_6O_3S$	S-adenosyl-L-methioninamine	1.96	0.10	0.042
7.39	230.015	230.019	$C_5H_{11}O_8P$	Ribulose-5-phosphate	2.07	0.50	0.053
13.19	607.070	607.082	$C_{17}H_{27}N_3O_{17}P_2$	UDP-N-acetyl-glucosamine	1.44	0.38	0.045
18.59	449.306	449.314	$C_{26}H_{43}NO_5$	Glycodeoxycholic acid	1.63	3.62	0.042
1.22	146.066	146.069	$C_5H_{10}N_2O_3$	Glutamine	1.04	0.95	0.053
1.24	89.046	89.048	$C_3H_7NO_2$	Alanine	1,80	0.86	0.042
1.23	119.056	119.058	$C_4H_9NO_3$	Threonine	1.61	1.19	0.042
1.23	119.056	119.058	$C_4H_9NO_3$	Homoserine	1.61	1.19	0.042
1.29	158.041	158.044	$C_4H_6N_4O_3$	Allantoin	1.62	0.45	0.01
15.36	154.024	154.027	$C_7H_6O_4$	2,3-dihydroxybenzoic acid	1.57	0.58	0.042
1.25	175.092	175.096	$C_6H_{13}N_3O_3$	Citrulline	1.48	0.17	0.053
7.16	172.010	172.014	$C_3H_9O_6P$	Sn-glycerol-3-phosphate	1.55	3.40	0.059
9.00	169.995	169.998	$C_3H_7O_6P$	Dihydroxy-acetone-phosphate	2.53	0.12	0.042
7.51	169.995	169.998	$C_3H_7O_6P$	D-glyceraldehdye-3-phosphate	2.19	0.10	0.053

Fold Change: Ratio of mean relative amount between the control and BPA groups; Adj P-value: Wilcoxon rank sum test P-value was adjusted for multiple testing using FDR.
