# Supplementary material for: Perinatal Bisphenol A Exposure Induces Chronic Inflammation in Rabbit Offspring via Modulation of Gut Bacteria and Their Metabolites
Source: mSystems. 2017 Oct 10;2(5):e00093-17. doi: 10.1128/mSystems.00093-17 (PMC5634791; doi:10.1128/mSystems.00093-17)
Supplement: TABLE S3 [file sys005172142st5.pdf]

Table S3: Receiver operator characteristic curve analysis of significant metabolites – targeted analysis

Biomarker	AUC	Best cutoff (normalized peak intensity)	Sensitivity	Specificity	Maximum of Youden Index
S-adenosyl-L-methioninamine	0.634	1665220	0.571	0.704	0.275
Ribulose-5-phosphate	0.598	4.18E+07	0.629	0.593	0.221
UDP-N-acetyl-glucosamine	0.614	4.27E+07	0.486	0.852	0.338
Glycodeoxycholic acid	0.605	6.50E+08	0.457	0.778	0.235
Glutamine	0.534	1.41E+09	0.457	0.778	0.235
Alanine	0.576	1.09E+08	0.457	0.778	0.235
Threonine	0.594	2.30E+08	0.457	0.778	0.235
Homoserine	0.594	2.30E+08	0.457	0.778	0.235
Allantoin	0.674	3.16E+07	0.600	0.778	0.378
Histidine	0.530	1.44E+08	0.429	0.778	0.206
2_3-dihydroxybenzoic acid	0.577	2.34E+07	0.800	0.482	0.282
Citrulline	0.583	9137220	0.686	0.519	0.204
Sn-glycerol-3-phosphate	0.535	2.09E+09	0.400	0.741	0.141
Dihydroxy-acetone-phosphate	0.601	3313770	0.629	0.593	0.221
D-glyceraldehdye-3-phosphate	0.594	2566500	0.714	0.519	0.233

AUC = area under the curve

*Sensitivity + specificity – 1
