# Supplementary material for: Perinatal Bisphenol A Exposure Induces Chronic Inflammation in Rabbit Offspring via Modulation of Gut Bacteria and Their Metabolites
Source: mSystems. 2017 Oct 10;2(5):e00093-17. doi: 10.1128/mSystems.00093-17 (PMC5634791; doi:10.1128/mSystems.00093-17)
Supplement: TABLE S4 [file sys005172142st6.pdf]

Table S4: Pathways that are significantly different between BPA-exposed and control offspring

Pathway	Total Compounds	Hits	Raw p-value	Adj p-value	Impact
		**COLON**			
Histidine metabolism	44	1	0.002	0.042	0.140
beta-Alanine metabolism	28	1	0.002	0.042	0.000
		**LIVER**			
Selenoamino acid metabolism	22	1	0.004	0.030	0.000
Taurine and hypotaurine metabolism	20	2	0.004	0.030	0.363
Amino sugar and nucleotide sugar metabolism	88	2	0.005	0.030	0.119
Nitrogen metabolism	39	4	0.005	0.030	0.008
Primary bile acid biosynthesis	47	1	0.006	0.030	0.008
Pentose and glucuronate interconversions	53	2	0.008	0.030	0.006
Galactose metabolism	41	2	0.008	0.030	0.001
Glycerolipid metabolism	32	2	0.008	0.030	0.000
Starch and sucrose metabolism	50	1	0.008	0.030	0.139
Ascorbate and aldarate metabolism	45	1	0.008	0.030	0.000
Pentose phosphate pathway	32	1	0.010	0.035	0.098
Aminoacyl-tRNA biosynthesis	75	7	0.011	0.035	0.056
Alanine, aspartate and glutamate metabolism	24	3	0.015	0.038	0.310
Arginine and proline metabolism	77	6	0.016	0.038	0.206
Pyrimidine metabolism	60	4	0.016	0.038	0.049
Purine metabolism	92	2	0.016	0.038	0.008
Fructose and mannose metabolism	48	1	0.022	0.038	0.041
Inositol phosphate metabolism	39	1	0.022	0.038	0.012
Glycolysis or Gluconeogenesis	31	1	0.022	0.038	0.000
Glycerophospholipid metabolism	39	1	0.022	0.038	0.000
Pyruvate metabolism	32	1	0.022	0.038	0.000
Nicotinate and nicotinamide metabolism	44	1	0.022	0.038	0.000
Cysteine and methionine metabolism	56	4	0.036	0.059	0.036

Adj p-value: Wilcoxon rank sum test raw p-value was adjusted for multiple comparisons (FDR).

Small p-value and large impact value indicate that the pathway is greatly influenced
